# Multiple Domestication Centers Revealed by the Geographical Distribution of Chinese Native Pigs

**DOI:** 10.3390/ani9100709

**Published:** 2019-09-21

**Authors:** Yuan Cai, Jinqiang Quan, Caixia Gao, Qianyun Ge, Ting Jiao, Yongbo Guo, Wangshan Zheng, Shengguo Zhao

**Affiliations:** 1 College of Animal Science & Technology, Gansu Agricultural University, Lanzhou 730070, China; 2 State Key Laboratory of Veterinary Biotechnology, Harbin Veterinary Research Institute, Chinese Academy of Agricultural Sciences, Harbin 150069, China; 3 College of Grassland, Gansu Agricultural University, Lanzhou 730070, China; 4 State Key Laboratory of Genetic Resources and Evolution, Kunming Institute of Zoology, Chinese Academy of Sciences, Kunming 650223, China

**Keywords:** pigs, mitochondrial DNA, phylogenetics, phylogeography

## Abstract

**Simple Summary:**

Phylogenetic analysis of Chinese native pigs was performed by screening for haplotypes inferred from a phylogenetic tree of pig mitochondrial DNA (mtDNA) sequences based on sequence-specific mutations. Our results suggest there are at least four domestication or expansion centers of Chinese native pigs, of which at least two domestication or expansion centers of Tibetan pigs are located in the Qinghai-Tibet Plateau and the intersection of the Hengduan Mountains (YSGH) of Yunnan, Sichuan, and Gansu provinces. The other two domestication or expansion centers are the Mekong River Basin in Yunnan Province and the middle and downstream regions of the Yangtze River.

**Abstract:**

Previous studies have shown that Southeast Asian pigs were independently domesticated from local wild boars. However, the domestication of Chinese native pigs remains a subject of debate. In the present study, phylogenetic analysis of Chinese native pigs was performed by screening for haplotypes inferred from a phylogenetic tree of pig mitochondrial DNA (mtDNA) sequences based on sequence-specific mutations. A total of 2466 domestic pigs formed 124 haplotypes and were assigned to four clades. Clade A comprised pigs distributed mainly in the Qinghai-Tibet Plateau and its surrounding areas; these pigs clustered into three groups. The pigs of clade B were mainly from the Mekong River Basin in Yunnan Province and had been exposed to genetic infiltration from European populations. Clade C comprised pigs mainly from the middle and lower reaches of the Yangtze River. The pigs of clade D were distributed mainly at the intersection of Yunnan, Sichuan, and Gansu provinces east of the Hengduan Mountains (YSGH). Compared with wild boar, at least three domestication centers and one expansion center of pigs in China were detected. Among the four centers detected, two were for Tibetan pigs and were in the Qinghai-Tibet Plateau and at the YSGH intersection, and the other two were in the Mekong River Basin in Yunnan Province and the middle and downstream regions of the Yangtze River.

## 1. Introduction

Pigs (*Sus scrofa*) are one of the oldest domesticated and most important socioeconomic livestock species in the world [1]. Human activities have changed the genetic structure of domestic pigs; as a result, the characteristics of this structure are important in the study of historical human activities [1,2,3,4,5]. According to archeological records, the history of pig breeding in China can be traced to more than 9000 years ago [6]. By the Neolithic Age, a large number of wild boars had been domesticated, which indicates that pig breeding had become an important part of the social economy by that time [7,8,9]. With the construction of a maternal phylogenetic map with mitochondrial DNA (mtDNA), a large amount of research has focused on analyzing the domestication process of global domestic pigs by mtDNA sequence data. Genetic and archeological evidence indicates that domestic and Asian pigs were independently domesticated from local wild boars and that there are significant differences in mtDNA between European and Asian domestic pigs [3,10,11,12]. Furthermore, independent pig domestication in China is supported by both genetic and archeological evidence [13]. Larson et al. [4] confirmed that the globally distributed wild boar originated on the island of Southeast Asia (ISEA), and subsequently distributed in Eurasia and multiple domestication centers in Europe and Asia, and then experienced different domestication events throughout the world. There may have been multiple domestication centers in Asia; native pigs in East Asia have experienced at least two domestication events. Additional studies demonstrated the influence of humans on the structure of domestic pig populations through the introgression of Asian germplasm into European pig breeds in the 18th century [2,4,14]. However, several domestication centers are unknown [15]. Geography, climate change, and large-scale human migration have affected the domestication of wild boar. China has diverse landforms and natural environments; hence, the domestication and distribution of native pig breeds are also more complicated. However, there is no genetic evidence to support a phylogenetic relationship between wild boar and domestic pigs in China, although independent pig domestication in China is supported by both genetic and archeological evidence [13,16,17]. However, previous studies on mtDNA in domestic pigs suggest that there are two domestication centers for Chinese native pig breeds [15], but didn’t include Tibetan pigs. Due to the lack of more exact geographic resolution of the mtDNA data for China, no conclusions can be drawn about multiple domestication centers in China [13]. Therefore, research on the origin and domestication of pigs in China is of great significance for germplasm resources protection. Native pigs have been classified into six types according to geographical location in China and physical characteristics: the (1) North China (NC) type, (2) lower Yangtze River Basin (LY) type, (3) Central China (CC) type, (4) South China (SC) type, (5) southwest (SW) type, and (6) plateau (PT) type [18,19,20]. To study the phylogeny and distribution of domestication events of indigenous pigs in China, we analyzed the mtDNA D-loop sequences of 2466 native pigs from China and further improved the phylogenetic tree and inferred domestication history and geographical distribution of Chinese native pigs.

## 2. Materials and Methods

### 2.1. Ethics Approval and Consent to Participate

All animal experiments were conducted according to the guidelines established by the ethics committee for the Care and Use of Laboratory Animals at Gansu Agricultural University.

### 2.2. Animals and Sample Collection

We collected samples from 842 native pigs from Sichuan, Tibet, Qinghai, Shandong, Gansu, and Yunnan provinces. The blood came from venous blood collection, the ear tissue came from the ear tags, and the muscle tissue was collected in the local slaughterhouses. The samples were collected in a 2 mL centrifuge tube, immersed in 70% absolute ethanol, and frozen at −80 °C for use. We combined these samples with 1646 sequences from GenBank, yielding a total of 2526 sequences (38 from wild boars, 22 from domestic boars, and 2466 from domestic pigs representing 87 breeds from 22 provinces).

### 2.3. DNA Extraction, Amplification, and Sequencing

Genomic DNA was extracted from blood, ear tissue, or muscle by the standard phenol/chloroform method. Primers were designed according to the Chinese wild boar complete sequence (EF545568.1) in GenBank, and DNA from 842 native pig samples was amplified by Polymerase Chain Reaction (PCR) to obtain the 431 bp mtDNA D-loop sequence; the amplification primers and conditions are described in Supplementary Materials Table S1 The original sequence data obtained by sequencing were manually edited by Chromas version 2.33 (http://www.technelysium.com.au/chromas.html), and the correspondence between the electrophoresis peak map and the bases was verified. The amplification and sequencing of the native pig mtDNA D-loop followed the protocols described in Supplementary Materials Table S1.

### 2.4. Phylogenetic Analysis

The genetic diversity of the six populations was calculated using DnaSP 5.0 (http://www.ub.edu/dnasp). In addition, neutrality tests, including Tajima’s D [21] and Fu and Li’s F [22], were implemented for each population in DnaSP 5.0 software. We selected the 124 haplotypes to construct a map of evolutionary relationships using median-joining networks by Network 5.0 (http://www.fluxus-engineering.com/sharenet.htm) and identified four clades: A, B, C, and D. To determine the specific domestication centers of clades A, B, C, and D, we assigned all individuals of these clades to 87 local breeds and matched them to corresponding locations on a map of China. A phylogenetic tree consisting of 124 mtDNA sequences of domestic pigs and 60 sequences of wild or domestic boars was constructed using the maximum likelihood method by MEGA 7.0 (http://www.megasoftware.net) [23]; bootstrap method was 1000, model/method was Kimura 2-parameter. We chose the African warthog (*Phacochoerus africanus*), which is different from European and Asian wild boars, as the outgroup.

### 2.5. Availability of Data and Materials

The data used in this study are being submitted to the GenBank database, and the datasets used and analyzed during the current study are available from the corresponding author upon reasonable request.

## 3. Results

### 3.1. Neutrality Test and Genetic Diversity of Chinese Native Pigs

We analyzed the genetic diversity of 2466 domestic pigs of 87 breeds from six geographic regions in China. The haplotype diversity (Hd) of the LY population was the highest among the populations, and that of the SW population was the lowest. A similar phenomenon was also detected for nucleotide diversity (Pi) and the average number of nucleotide differences (K). Under the hypothesis of selective neutrality and population equilibrium, Tajima’s D and Fu’s Fs test values tend to be negative under an excess of recent mutations, which is regarded as evidence of population expansion [21,22]. Tajima’s D and Fu’s Fs tests of six populations showed that only the PT population values were negative, and the difference was significant for both tests, indicating that the expansion of the PT population was greater than that of the other groups (Table 1). The NC and SW populations also showed significant signs of expansion based on Fu’s Fs test; the other populations showed no significant signs of expansion (Supplementary Materials Table S2).

### 3.2. Construction of the Chinese Native Pig Phylogenetic Relationship Network

We reconstructed the phylogenetic relationships of 87 native breeds by using 2466 domestic pigs. Relative to European wild boar (AF304201.1), Chinese wild boar (EF545568.1) has 11 mutations in the 431 bp mtDNA D-loop, which comprise 10 transformations and 1 indel locus at positions 15,459 (A/G), 15,478 (T/C), 15,497 (G/A), 15,550 (G/A), 15,514 (C/T), 15,583 (T/C), 15,609 (T/C), 15,645 (C/T), 15,692 (C/T), 15,759 (C/T), and 15,506 (C). Similarly, we compared the mtDNA D-loop of Chinese wild boars and native pigs and found mutations at 4 sites: 15,550 (T/C), 15,609 (C/T), 15,610 (T/C), and 15,675 (C/T). Within Chinese native pigs, we found two important mutations, namely, T transformed to C at the 15,610 and 15,648 positions in the mtDNA D-loop, and the Chinese native pigs were differentiated by the 15,610 and 15,648 sites. Individuals without mutations at these two sites were assigned to clade D, those with only a mutation at the 15,610 site were assigned to clade C, those with a mutation at the 15,648 site were assigned to clade B, and those with mutations at both the 15,610 and 15,648 sites were assigned to clade A (Supplementary Materials Figure S1).

**Figure 1:**
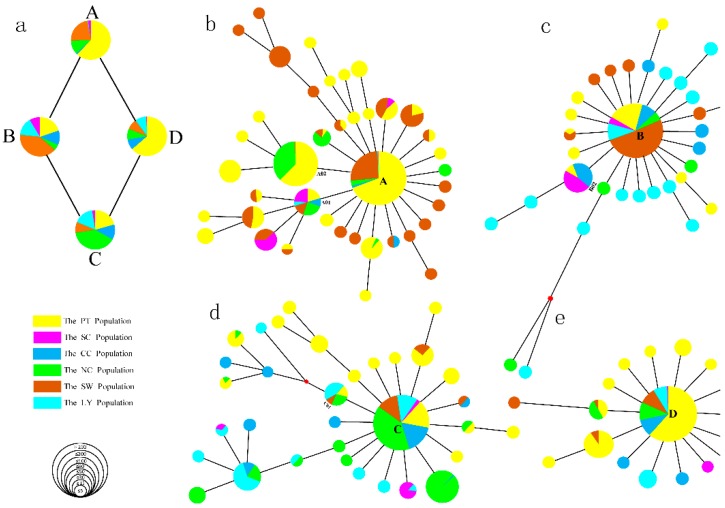
(**a**) A total of 2466 mitochondrial DNA (mtDNA) samples of native pigs representing 124 haplotypes were assigned to four clades (clades A, B, C, and D) in a reduced median network. (**b**) Clade A distribution trend; (**c**) clade B distribution trend; (**d**) clade C distribution trend; (**e**) clade D distribution trend. Most of the samples were from pigs from six different geographical regions of China: North China (NC); the lower Yangtze River Basin (LY); Central China (CC); South China (SC); the southwest (SW); and the plateau (PT). The different regions are represented by different colors. Circle size indicates the number of populations, except in (**a**) A red dot represents a single mutation site.

The 2466 individuals formed 124 haplotypes according to different mutation sites in the mtDNA D-loop (Supplementary Materials Table S3). Clade A contains 1107 individuals from 53 local pig breeds, clade B contains 339 individuals from 60 local pig breeds, clade C contains 591 individuals from 63 local pig breeds, and clade D contains 429 individuals from 51 local pig breeds. Clades A, B, C, and D have their own mutation characteristics and exhibit a star-shaped radiation structure. The PT population contains 64 haplotypes, including 40 unique haplotypes and 24 shared haplotypes, and the SC population contains the fewest haplotypes, with only 2 unique haplotypes and 10 shared haplotypes. We also found that haplotypes A, B, C, D, and A01 were shared among six populations (Supplementary Materials Table S3). A phylogenetic network was reconstructed based on the relationships of haplotypes and variation among individuals using 124 haplotypes (Figure 1). The network mainly forms four clades (i.e., A, B, C, and D) and each clade is surrounded by a star-like pattern, consistent with recent population expansion and analogous to the pattern seen in cattle [24,25].

### 3.3. Geographical Distribution of Chinese Native Pigs

**Figure 2:**
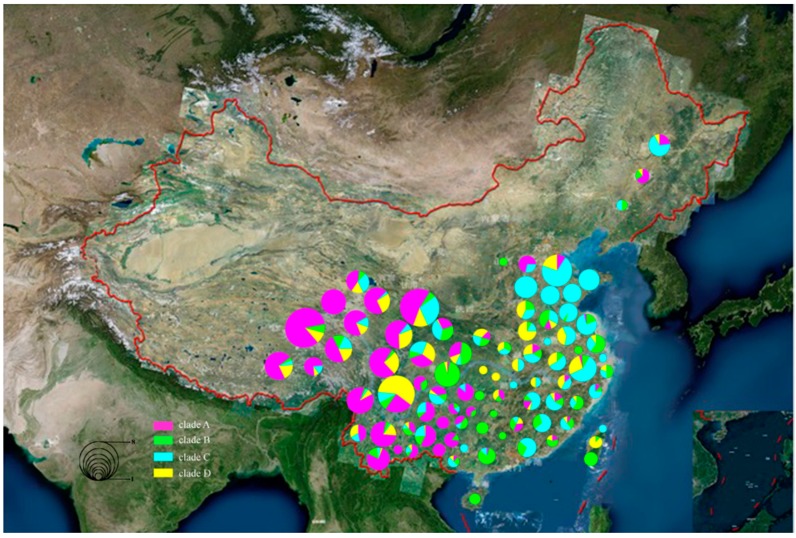
According to the origin of each individual in each clade, 2466 pigs representing 87 local breeds are shown on a map of China. Different clades are represented by different colors. Circle size indicates the number of individuals; for example, circle 1 contains one individual, circle 2 contains ≤5, circle 3 contains ≤10, circle 4 contains ≤30, circle 5 contains ≤50, circle 6 contains ≤100, circle 7 contains ≤150, and circle 8 contains >150.

To predict the domestication area and expansion process of clades A, B, C, and D, we assigned each individual in these clades to a position on a map of China (Figure 2). We found an interesting phenomenon in which clade A was mainly distributed in the Qinghai-Tibet Plateau and there were two different directions of distribution: some individuals flowed to Qinghai and Gansu provinces and expanded to the northwest in China. The others expanded to Yunnan Province and other places in southwestern China. Clade B is mainly distributed in the southern Yangtze River area and expanded from Sichuan Province to the southeast of China. Clade C is mainly distributed in the middle and downstream regions of the Yangtze River in China and expanded from Zhejiang to Fujian Province. Clade D is mainly distributed upstream of the Yangtze River at the Hengduan Mountains at the junction of Gansu, Yunnan, and Sichuan Provinces.

We used six colors to represent populations of the six geographic types and marked the populations in clades A, B, C, and D. The PT population is mainly concentrated in clades A and D, the SW population is mainly concentrated in clade B, and the NC population is mainly concentrated in clade C. Therefore, we conclude that three populations (the PT, SW, and NC populations) are mainly distributed in four domestication centers (Figure 1). To determine more precisely the locations of the domestication centers, we assigned all individuals of the four clades to 87 breeds and traced the locations of the domestication centers according to the background of each breed (Figure 2).

Based on analysis of the genetic diversity and neutrality of each population in clade A, only the PT and SW populations differ significantly in Tajima’s D and Fu and Li’s F test, indicating that these populations have expanded. Simultaneously, we analyzed clade A and found that it was mainly composed of Tibetan pigs distributed in the Qinghai-Tibet Plateau (62.15%) and the SW population of native pigs (27.1%).The Tibetan pigs in clade A are mainly distributed in Tibet (43.6%) and Gansu (23.84%) provinces, and the SW population is mainly distributed in the local varieties of the upstream Mekong River Basin in Yunnan Province.

### 3.4. Phylogenetic Tree of Chinese Native Pigs

**Figure 3:**
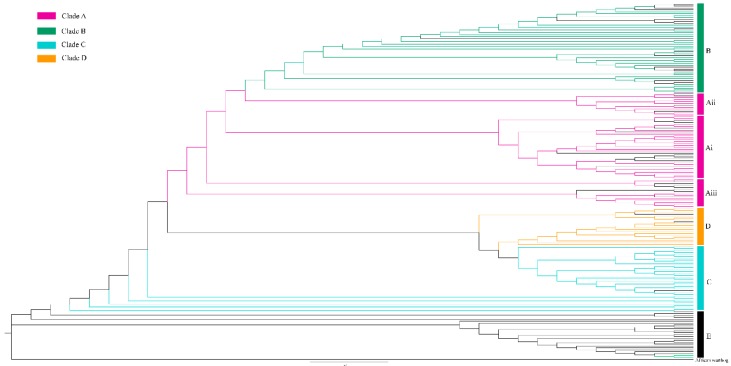
Phylogenetic tree of Chinese native pigs constructed with 124 haplotypes and 60 reference sequences by the maximum likelihood method, with the African warthog (DQ409327.1) as the outgroup. Different clades are represented by different colors. Clade A is clustered into 3 groups (Ai, Aii, and Aiii). Clades B, C, and D each cluster into one group. E represents European wild boars and domestic pigs.

We reconstructed the phylogenetic tree of Chinese native pigs by using the mtDNA D-loop, the African warthog as the outgroup, and 60 published mtDNA sequences as a reference. We found results similar to those for the network. In the phylogenetic tree, the Chinese pig sequences mainly clustered into seven groups (Figure 3 and Supplementary Materials Figure S2). Clade A is divided into three groups (Ai, Aii, and Aiii), clade B clustered into the European group, and clades C and D, the remaining haplotypes, belong to a mixed group, making it difficult to accurately distinguish or locate them.

## 4. Discussion

### 4.1. Multiple Domestication Centers for Chinese Native Pigs

The domestication process and expansion pattern of domestic pigs help in the study of human migration behavior, and the domestication and expansion of pigs in Southeast Asia have always been a source of debate among many scholars. The mtDNA of European and Asian pigs differentiated before the purported domestication of pigs, supporting the independent origins of European and Asian domestic pigs [3,26]. Previous studies by Larson et al. suggested that globally distributed pigs originated on the ISEA and subsequently expanded to Europe and Asia, thereafter forming several domestication centers in Eurasia [4]. Subsequent research identified six domestication centers of native pigs in East Asia [13], and Wu et al. [15] identified the Mekong River Basin in Yunnan Province as one of the domestication centers of Chinese native pigs. Lu’s study of the history of wild boar domestication in Southeast Asia suggests that after thousands of years of hunting and gathering, a major biocultural change took place in the early Holocene, during which the population of East Asia domesticated various plants and animals, including pigs [27]. This process occurred at least once in the Yellow River Basin, and domestication near the Yellow River occurred as early as 10,000 Before Christ (BC) [27]. Wu et al. [15] suggested that the limited regional distributions of haplogroups D1 (including its subhaplogroups), D2, D3, and D4 of domestic pigs indicate at least two different in situ domestication events and that the domestication of East Asian pigs mainly occurred in the Mekong region and the middle and lower reaches of the Yangtze River. Yang et al. [28] studied the origins of Asian pig breeds and identified a new origin center for domestic pigs in the Tibetan highlands. We use different colors to represent different populations (Figure 1), and the results show that the PT population is mainly distributed in clades A and D, and the SW and NC populations are divided into clades B and C, respectively. The PT population accounts for 62.15% of clade A, in which Tibetan pigs from Tibet are dominant (43.60%). Therefore, we speculate that clade A represents a domestication event that occurred on the Tibetan Plateau. Simultaneously, we found that clades B and C likely represent domestication events in the areas surrounding the Mekong River Basin in Yunnan Province, the middle and lower reaches of the Yangtze River, and clade D likely represents expansion events in the intersection of Yunnan, Sichuan, and Gansu Provinces east of the Hengduan Mountains (YSGH), respectively.

### 4.2. At Least Two Domestication Centers of Tibetan Pigs

Tibetan pigs are mainly distributed in the Qinghai-Tibet Plateau and its surrounding areas. Previous studies have shown that the Qinghai-Tibet Plateau was an independent domestication center for Tibetan pigs [28]. This result is consistent with our findings (such as clade A), and we have also discovered an interesting phenomenon: there are significant differences between Tibetan pigs (clade D) and Tibet Tibetan pigs (clade A), and they should be from different domestication centers. This area is located exactly at the YSGH intersection (Figure 2). Located in the Hengduan Mountains in the south of the Qinghai-Tibet Plateau in northwestern Yunnan, the intersection is a unique area because of its alpine landforms and evolution, and it is one of the most biologically diverse areas in the world [29,30]. We speculate based on lower genetic diversity of clade D that because the Hengduan Mountains act as a natural barrier separating Tibet from the interior, Tibetan pigs expanded at the YSGH intersection. The Tibet Tibetan pig was domesticated earlier, but it did not expand to the mainland due to geographical restrictions. However, Tibetan pigs at the YSGH intersection were domesticated later than Tibet Tibetan pigs but expanded to the southeast along the Yangtze River. Although Gansu Tibetan pigs (A02) belong to clade A, they are distinct from other Tibetan pigs in the mtDNA D-loop region, and the genetic diversity of Gansu Tibetan pigs is much higher than that of other Tibetan pigs. Larson’s research suggested that the Gansu pigs represented an independent haplotype [4]. Based on analysis of the ancient DNA of pig fossils from the upstream of Yellow River Basin, Wang et al. [31] concluded that there were domestication centers of domestic pigs in the Yellow River Basin, although the specific locations were not determined. We speculate that Gansu Province, which is located in the upstream of Yellow River Basin, may have been a domestication or expansion center for Tibetan pigs, but there is still not enough evidence of ancient DNA and archeology. More detailed phylogenetic analyses are needed to test this possibility.

### 4.3. Domestication and Distribution of Other Native Pigs

We further analyzed clade B and found that it mainly consisted of the LY population (Figure 1) and was mainly located south of the Yangtze River (Figure 2). Both genetic diversity and Tajima’s D indicate that the LY population has expanded (Supplementary Materials Table S2). In clade B, we found that two haplotypes, namely, B01a01b and B01a01a, differed greatly from other haplotypes and were closely related to European populations based on the phylogenetic tree. According to historical records, in the 18th century, Chinese miniature pigs were brought to the European continent [2,4,14]. The main breeds in clade B are minipigs from the southeastern region in China, including Wuzhishan minipigs, Xiang minipigs, and other minipig breeds. Therefore, we considered clade B to be a domestication center for minipigs, mainly distributed in the southeastern region of China. The combination of genetic material in Europe and Asia gave rise to modern European pig breeds. We further identified the breeds in clade B and obtained consistent results.

Clade C is mainly distributed in the middle and lower reaches of the Yangtze River. According to Wu’s study, the middle and lower regions of the Yangtze River were one of domestication areas in Southeast Asia [15]. We further analyzed the breeds in clade C and found that they were mainly Taihu breeds. The Taihu breeds are world-famous, high-fertility, native breeds that have been widely introduced throughout the world as hybrid parents in recent years. Therefore, we speculate that clade C represents a domestication center of the Taihu breeds in the middle and lower reaches of the Yangtze River.

### 4.4. Phylogenetic Relationships of Chinese Native Pigs

The members of the Ai group, Tibetan pigs, and D2 wild boars clustered together in one unit in clade A in the phylogenetic analysis, and all were Tibetan Plateau pigs (Figure 3 and Supplementary Materials Figure S2). These results are consistent with our speculation above [15,32]. However, the Aii group and the Ai group have similar results but are assigned to different evolutionary branches. Based on the results presented in Figure 1 and Figure 2, we speculate that Gansu Province, located in the upper and middle reaches of the Yellow River, is also a domestication or expansion area for Tibetan pigs. Furthermore, the Aiii group was separately assembled into one unit, so we speculate that the Diqing Tibetan pigs of Yunnan Province differ from the pigs of the Tibetan Plateau population. However, the D group and the Mekong River Basin, Sichuan, and Gansu wild boars clustered together [15], suggesting that native pigs from this area may have arisen from the same domestication or expansion event. This area is located at the intersection of Yunnan, Sichuan, and Gansu provinces east of the Hengduan Mountains (YSGH). Similarly, we found that the B group and the Southeast Asian pigs clustered together [15]. Previous studies have shown that European and Southeast Asian populations were domesticated independently, and the present findings are consistent with this observation. European wild boars and domestic pigs clustered into group E, and several individuals from clade B are mixed in with them, suggesting their relatedness [33]. Previous studies have confirmed that in the 18th century, native pigs of Southeast Asia entered Europe, and genetic infiltration occurred [2,4,14]. Furthermore, group C and pigs from the middle and lower reaches of the Yangtze River (MDYZ) clustered together [20].

## 5. Conclusions

In previous studies, many researchers have speculated that there were at least two domestication centers of Chinese native pigs. According to our research, there may be at least three domestication centers and an expansion center of domestic pigs in China. Native pigs are mainly distributed in the middle and downstream regions of the Yangtze River, the Mekong River Basin in Yunnan Province, the YSGH intersection, and the Qinghai-Tibet Plateau. For the Tibetan pigs, at least one domestication center and one expansion center were located in the Qinghai-Tibet Plateau and the YSGH intersection. Gansu Province may also be a Tibetan pig domestication center, but this speculation requires further confirmation.

## Figures and Tables

**Table 1:** Neutrality test and genetic diversity metrics of six populations of Chinese native pigs.

Population	Hd ± SD	Pi	K	Tajima’s D	Fu’s Fs test
NC (413)	0.817 ± 0.012	0.00412	1.794	−1.30431	−2.57811 *
LY (189)	0.872 ± 0.011	0.00554	2.411	−1.40856	−0.74067
CC (133)	0.812 ± 0.020	0.00396	1.723	−1.02415	−2.22600
SC (86)	0.861 ± 0.020	0.00512	2.228	0.50134	0.14430
SW (494)	0.802 ± 0.012	0.00355	1.545	−1.54150	−3.25334 *
PT (1151)	0.808 ± 0.009	0.00403	1.752	−1.82777 *	−2.85157 *

The size of each populations in parentheses; Hd: haplotype diversity; SD: standard deviation; Pi: nucleotide diversity; K: average number of nucleotide differences; *: significant difference (*p* < 0.05).

## Supplementary Materials

The following are available online at https://www.mdpi.com/2076-2615/9/10/709/s1, Table S1. Information on the amplification primers, conditions and procedures. Table S2. Distribution of genetic diversity and neutrality test results for native pig breeds in China. Table S3. Information on the individual sequences used in this study and on the individuals and haplotype frequencies distributed among the four clades. Figure S1. Differences between Chinese native pigs and European wild boars. Figure S2. Detailed phylogenetic tree of Chinese native pig breeds, wild boars, and other domestic pigs.
