# Supplementary material for: Multiple Domestication Centers Revealed by the Geographical Distribution of Chinese Native Pigs
Source: Animals (Basel). 2019 Sep 21;9(10):709. doi: 10.3390/ani9100709 (PMC6827149; doi:10.3390/ani9100709)
Supplement: Supplementary file 1 [file animals-09-00709-s001.zip › Figure S2.docx]

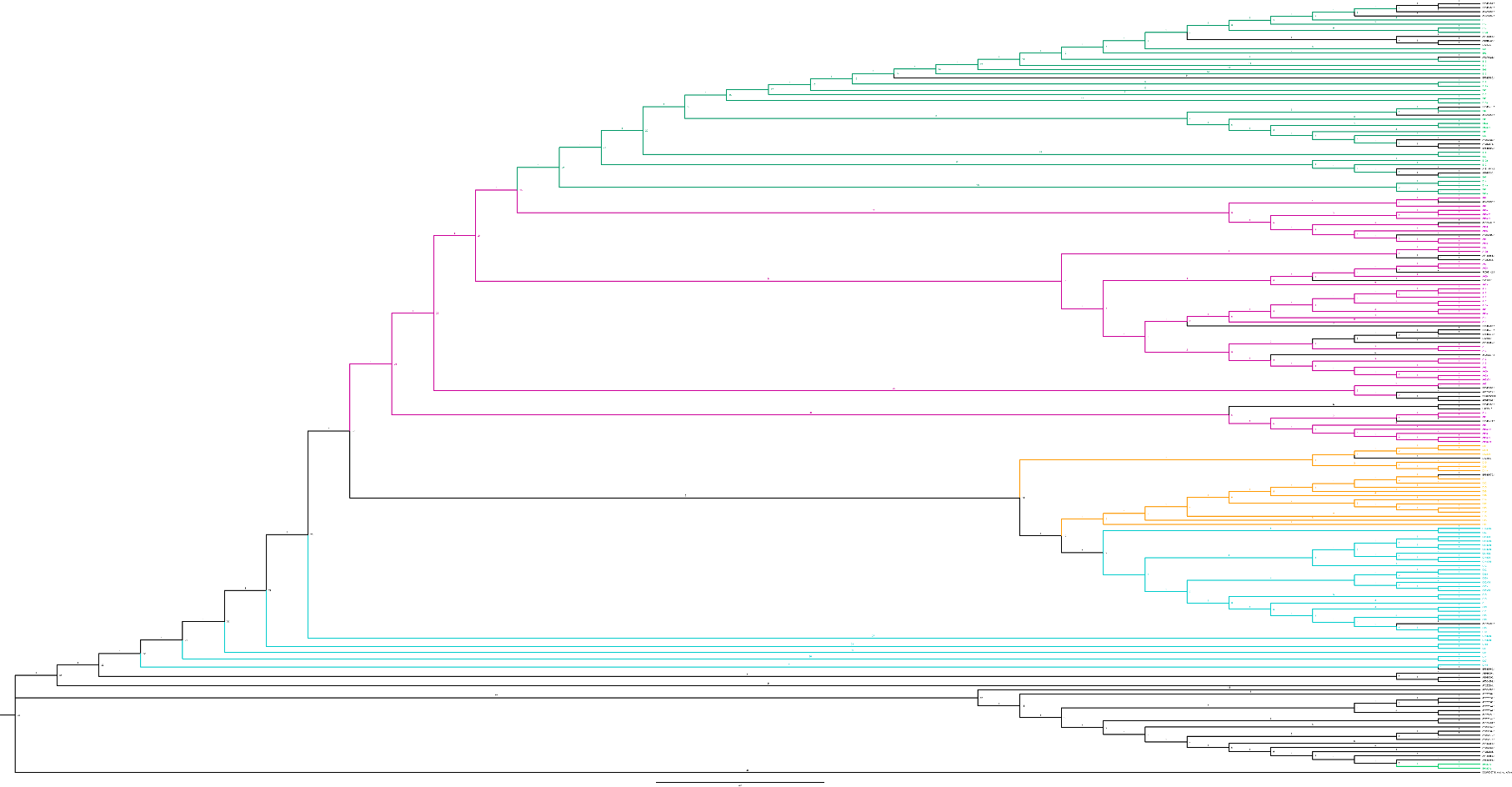


**Figure S2.** Phylogenetic tree of Chinese native pigs constructed with 124 haplotypes and 60 reference sequences by the maximum likelihood method, with the African warthog (DQ409327.1) as the outgroup. Different clades are represented by different colors. Clade A is clustered into 3 groups (Ai, Aii and Aiii), Clade B, clades C and D are each clustered into one group, E represented European wild boars and domestic pigs. This figure is a detailed diagram of Figure 3.
