# Supplementary material for: Multiple Domestication Centers Revealed by the Geographical Distribution of Chinese Native Pigs
Source: Animals (Basel). 2019 Sep 21;9(10):709. doi: 10.3390/ani9100709 (PMC6827149; doi:10.3390/ani9100709)
Supplement: Supplementary file 1 [file animals-09-00709-s001.zip › Table S1.docx]

**Table S2**. Information of amplification primer

| **Primer** | **5’ - 3’** | **Size(bp)** | **Tm(°C)** | **Reference** |
| --- | --- | --- | --- | --- |
| Df | CCAAAAACAAAGCAGAGTGTAC | 436 | 56 | Zhang J, 2016 |
| Dr | CGTTATGAGCTACCGTTATA |  |  |  |

Amplification procedures: The PCR reaction mixture consisted of 25 μL, containing 12.5 μL2× Eco Taq PCR Supermix containing 1 U Taq polymerase, 500 mΜ dNTPs, and 10×Taq buffer (Beijing TransGen Biotech Co., Ltd., China), 0.1 μg of template DNA, 0.4 μL of 10 pmol/mL of each primer and 11.6 μL of ddH_2_O. The cycling conditions were initial denaturation at 94 °C for 5 min, followed by 33 cycles of 94 °C for 30 s, 56 °C for 30 s and 72 °C for 30 s, and a final extension for 5 min at 72 °C .Amplified DNA fragments were purified following agarose gel electrophoresis and sequenced using the ABI 3130 DNA sequencer (Applied Biosystems, Foster City, CA, USA).
