# Supplementary figures and images for: Multiple Domestication Centers Revealed by the Geographical Distribution of Chinese Native Pigs
Source: Animals (Basel). 2019 Sep 21;9(10):709. doi: 10.3390/ani9100709 (PMC6827149; doi:10.3390/ani9100709)

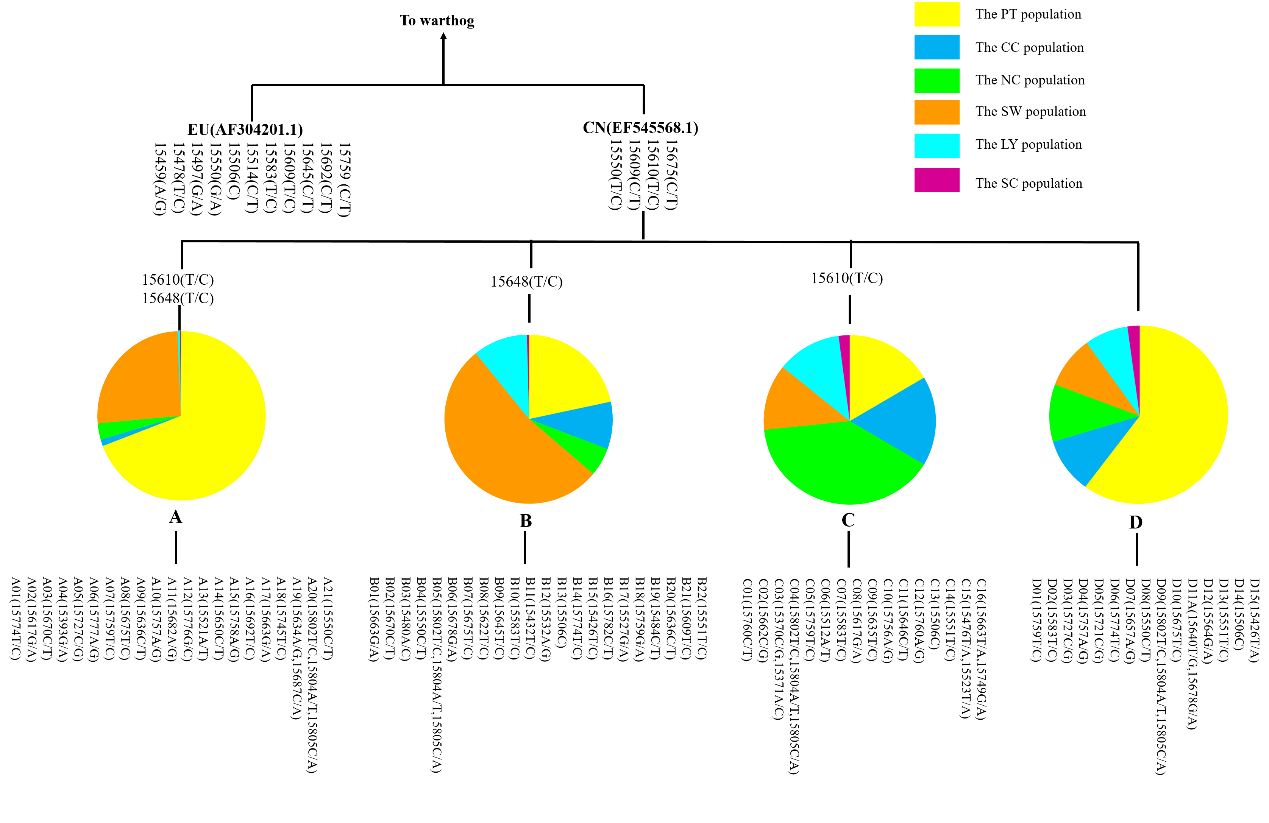


**Figure S1**. Comparison of differences between Chinese native pigs and European wild boars

Supplement: Supplementary file 1 [file animals-09-00709-s001.zip › Figure S1.docx]
